# Triboelectric energy harvesting with surface-charge-fixed polymer based on ionic liquid

**DOI:** 10.1080/14686996.2018.1448200

**Published:** 2018-03-23

**Authors:** Chikako Sano, Hiroyuki Mitsuya, Shimpei Ono, Kazumoto Miwa, Hiroshi Toshiyoshi, Hiroyuki Fujita

**Affiliations:** a Institute of Industrial Science, The University of Tokyo, Tokyo, Japan; b Saginomiya Seisakusho Inc., Sayama, Japan; c Central Research Institute of Electric Power Industry, Yokosuka, Japan

**Keywords:** Triboelectric energy harvesting, triboelectric nanogenerator, ionic liquid, electrical double layer, 50 Energy Materials, 206 Energy conversion / transport / storage / recovery

## Abstract

A novel triboelectric energy harvester has been developed using an ionic liquid polymer with cations fixed at the surface. In this report, the fabrication of the device and the characterization of its energy harvesting performance are detailed. An electrical double layer was induced in the ionic liquid polymer precursor to attract the cations to the surface where they are immobilized using a UV-based crosslinking reaction. The finalized polymer is capable of generating an electrical current when contacted by a metal electrode. Using this property, energy harvesting experiments were conducted by cyclically contacting a gold-surface electrode with the charge fixed surface of the polymer. Control experiments verified the effect of immobilizing the cations at the surface. By synthesizing a polymer with the optimal composition ratio of ionic liquid to macromonomer, an output of 77 nW/cm^2^ was obtained with a load resistance of 1 MΩ at 1 Hz. This tuneable power supply with a μA level current output may contribute to Internet of Things networks requiring numerous sensor nodes at remote places in the environment.

## Graphical abstract

**Figure:**
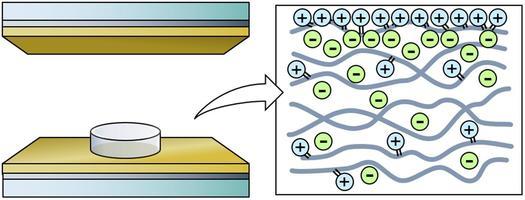

## 1. Introduction

With the increase in environmental issues caused by the use of limited traditional resources, renewable and clean forms of energy have been attracting worldwide attention. Given the increasing ubiquity of distributed sensor networks, known as the Internet of Things (IoT), there is a growing need for power supplies for the numerous sensor nodes which are placed at remote places in the environment. Since the power required by the distributed network devices is decreasing to the sub-mW level [1] or due to their growing efficiency, energy harvesters have become a very attractive solution to eliminate the necessity of power-cable connections or batteries that require regular replacement. Among all the ambient energy sources such as light, wind, heat, mechanical vibration, and biological substances, we focus on mechanical vibration that exists everywhere at any time [2], i.e. day and night, regardless of weather conditions.

Previous research works adopted different transduction mechanisms to convert mechanical energy to electricity [3,4]: electromagnetic induction [5–7], piezoelectricity [8–11], electrostatic induction [12,13], and triboelectricity [14,15]. Triboelectric energy harvesters utilize the electric charge separation and induction when two materials are brought into contact and separated from each other by an external force. These devices are characterized by both simple fabrication and excellent mechanical reliability.

We have investigated the application of ionic liquids to vibrational energy harvesters [16] and developed a method to form an ionic liquid polymer with cations immobilized at the surface [17]. The surface-charge-fixed polymer is synthesized by solidifying a polymer matrix mixed with an ionic liquid in the presence of an external electric field. When placed on top of a gold electrode, an electrical output is observed as the counterpart gold electrode makes contact with and separates from the polymer. In this paper, the energy harvesting performance of this triboelectric vibrational energy harvesting system utilizing the surface-charge-fixed polymer is described.

## 2. Triboelectric energy harvesting systems

The functional principle of triboelectric energy harvesting systems is a combination of contact electrification and electrostatic induction. Pair of different materials, typically dielectrics, are situated opposing each other with a gap distance that is varied upon the application of mechanical force. Different classifications of triboelectric energy harvesters exist depending on the direction of the mechanical force, as well as the relationship between the dielectric materials and the electrodes [14,15]. With regards to the direction of the force, our harvester can be categorized as a ‘contact-mode’ device [18–26] since the input force is perpendicular to the material surface causing the contact and separation of materials, rather than a ‘sliding/bending-mode’ device [27–33].

A typical contact-mode triboelectric energy harvester with a pair of electrode-attached-dielectrics features two main modes of operation. At the initial contact, triboelectric polarization occurs at the interface between the two dielectrics. In the continuous contact-separation cycles, this charge polarization is maintained, and electrostatic induction triggers the transfer of charges between the electrodes.

In addition, it is known that the intrinsic dipole moment of the dielectric material affects the electrical output of triboelectric energy harvesters [34]. The output voltage of a polyvinylidene difluoride (PVDF)-based triboelectric energy harvester was either enhanced to 240% or reduced to 70% depending on the direction of the polarization altering the surface potential level of the PVDF film, thus modulating the surface charge transfer between the PVDF film and the aluminum electrode.

In our energy harvesting system utilizing a surface-charge-fixed polymer, we propose a similar but distinct mechanism based on contact electrification. The immobilized cations increase the amount of contact electrification by altering the surface potential level of the polymer. However, the triboelectric charges are discharged through the polymer during the separated phases since there are mobile anions present in the polymer as well.

## 3. Fabrication of surface-charge-fixed polymer

The surface-charge-fixed polymer consists of three components: a base material, an ionic liquid, and a photoinitiator. The base material is made up of macromonomers featuring a crosslinking functional group (TA210: DKS Co. Ltd., Kyoto, Japan). The cations in the ionic liquid ((2-Methacryloyloxyethyl)trimethylammonium bis(trifluoromethanesulfonyl)imide) possess unsaturated bonds corresponding to those present in the macromonomers. Typically, an ionic liquid in contact with an electrode forms a thin (~1 nm) electrical double layer (EDL) which leads to a large capacitance (~10 μF/cm^2^) when a voltage within a certain threshold, known as the electrochemical window, is applied across it [35]. The electrochemical window of this ionic liquid material is −1.3 to 2.9 V. The initiator allows polymerization of the solution after mixing appropriate amounts of constituent materials and exposing to UV light.

**Figure 1.**
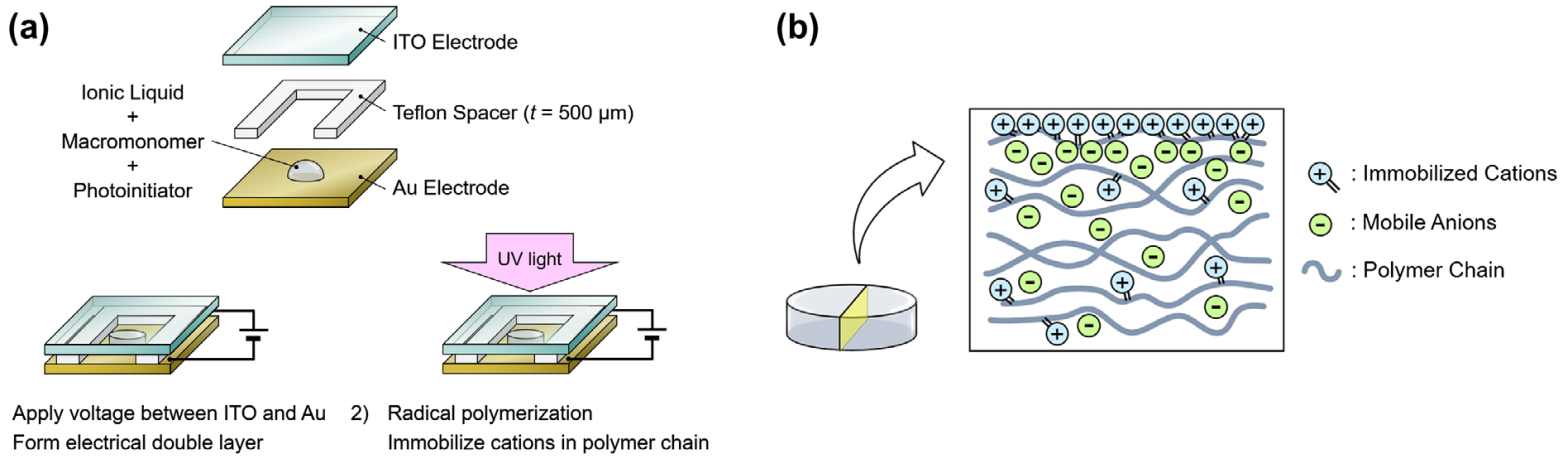
(a) Schematic of fabrication process of surface-charge-fixed polymer. (b) Schematic of internal structure of fabricated polymer.

First, the ionic liquid, macromonomers, and the initiator was mixed and a droplet of the solution was placed between a pair of transparent indium tin oxide (ITO) and gold electrodes with a spacer of 500 μm to define the height of the polymer (Figure 1(a)). We made the polymers with ionic liquid to macromonomer mixing ratios ranging from 0, 1, 5, 10, 15, to 20 wt%. The typical diameter of the polymer was 5 mm. Then, a bias voltage of 3 V was applied across the electrodes in order to form an EDL at the electrode/ionic-liquid interface. Maintaining the bias voltage, the sample was exposed to UV light to crosslink the macromonomers into a polymer and to immobilize the cations on the polymer chains. The current between the electrodes was monitored throughout this procedure with a source measure unit (2280S-60-3: Keithley Instruments, Cleveland, OH, USA). The UV exposure was considered to be complete when the current decreased to the μA level, which typically took 15 min. All procedures were conducted at room temperature. Figure 1(b) is a schematic of the internal structure of the fabricated polymer with mobile anions while the cations are immobilized in the polymer especially within the EDL region.

## 4. Energy harvesting measurement setup

**Figure 2.**
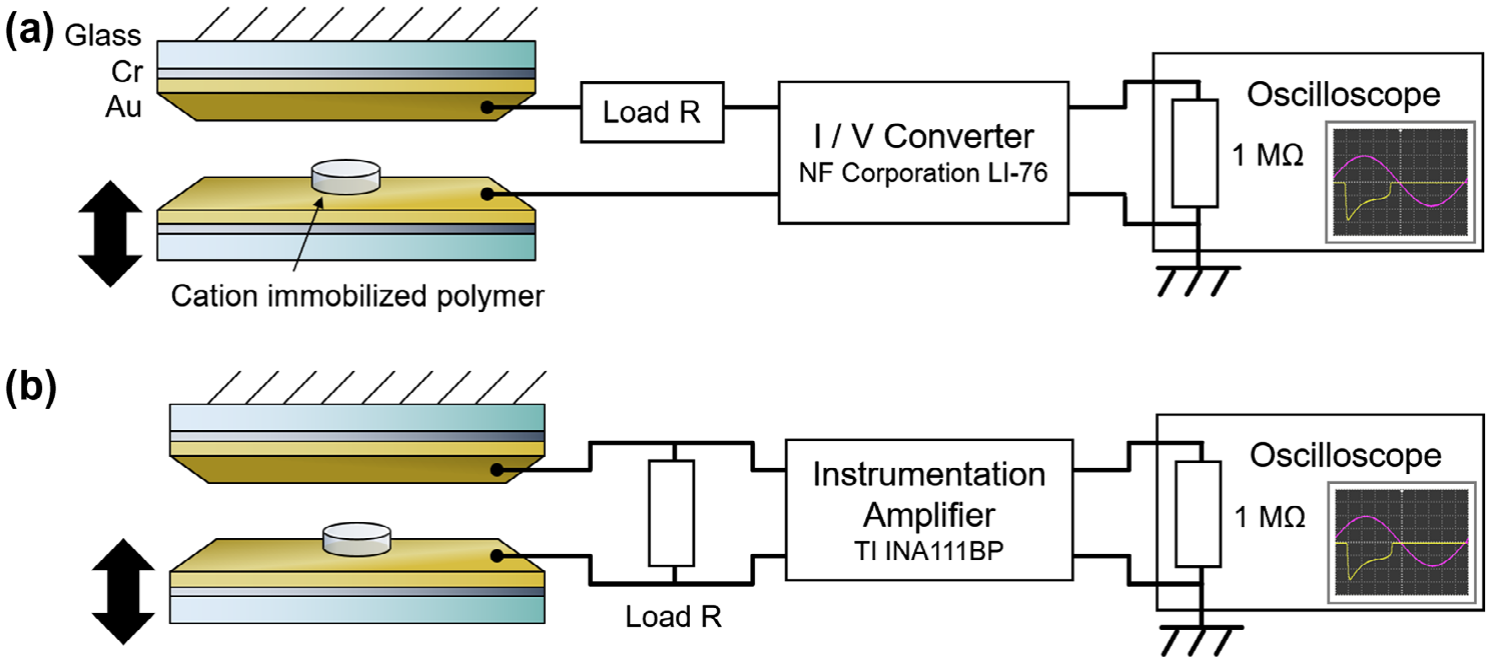
(a) Schematic of device structure and current measurement setup using a current input preamplifier. (b) Schematic of device structure and voltage measurement setup using an instrumentation amplifier.

The fabricated polymer was placed between a pair of electrodes with the cation immobilized surface on the top. The electrodes were 15-nm-Cr/100-nm-Au layers on glass. Due to its chemical stability, gold was chosen as an electrode material to avoid any chemical degradation of the electrode that could inhibit or interfere with the triboelectric effect. Chromium acts as an intermediate adhesion promoter to enable the deposition of a mechanically stable gold film on glass. The lower electrode was mechanically driven up and down in a sinusoidal displacement using a vibration generator causing the polymer to cyclically contact with and separate from the upper electrode during which the electrical output was measured. The experimental setup for the current measurement with an I/V converter (LI-76: NF Corporation, Yokohama, Japan) is shown in Figure 2(a). Also, the experimental setup for the voltage measurement with an instrumentation amplifier (INA111BP: Texas Instruments Inc., Dallas, TX, USA) is shown in Figure 2(b). All voltage outputs were measured using an oscilloscope (TDS2024B: Tektronix, Inc., Beaverton, OR, USA) with a sampling rate of 2500 samples per second. For stability, all waveform data shown in this manuscript was obtained several tens of contact-separation cycles after the vibration was initiated in order to more accurately represent the long-term performance of the device. The wave profiles for all samples exhibited the largest power generation during initial contact, decaying within several cycles and saturating to a stable wave profile.

## 5. Energy harvesting measurement results and discussion

**Figure 3.**
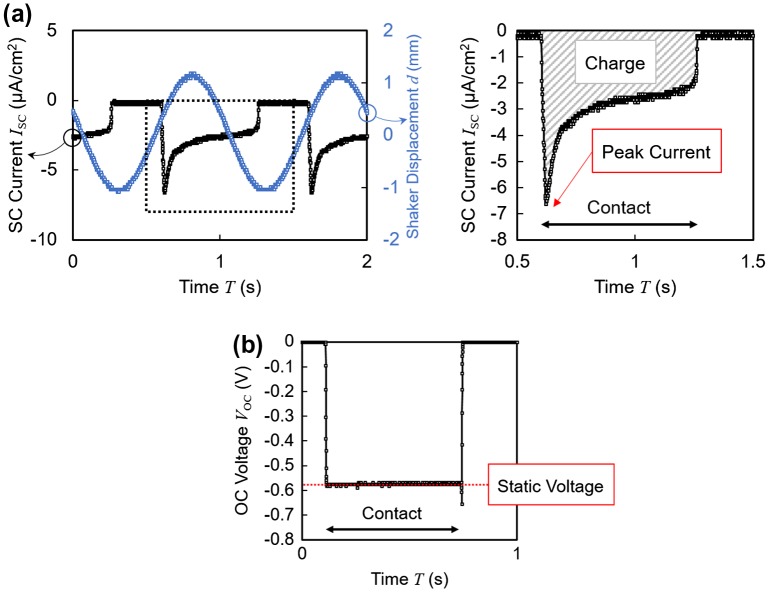
(a) Typical short circuit (SC) current wave profile. The sinusoidal shaker displacement is also included in the left panel to show the phase relationship between the current and the mechanical excitation. (b) Typical open circuit (OC) voltage wave profile.

Figure 3(a) and (b) shows typical waveforms of the short circuit current and open circuit voltage outputs obtained from the energy harvesting routine. In both cases, the unipolar outputs were detected only when the electrode was in direct contact with the polymer. As shown in Figure 3(a), short circuit currents exhibited an initial peak followed by an exponential decay of *τ* ~ 0.4 s. In the following experimental results, the current integrated over the duration of contact will be referred to as the amount of transferred charge. In contrast, as shown in Figure 3(b), open circuit voltages exhibited rather flat profiles. The stable voltage level achieved during contact is defined as the ‘static voltage.’

**Figure 4.**
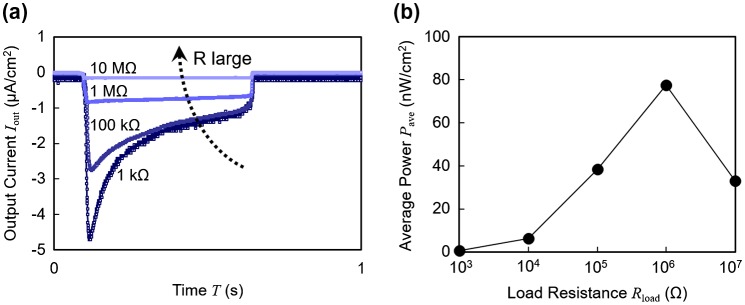
(a) Output current wave profiles with varying load resistance. (b) Average power plotted with respect to load resistance.

The output current and the output power of a 10 wt% ionic liquid polymer are shown in Figure 4(a) and (b), respectively, as a function of load resistance. In Figure 4(a), the wave profile exhibited a sharp initial peak in the current at lower resistance values while higher loads yielded flatter profiles. Higher resistances limited the rate of the charge transfer between the electrodes, leading to wave profiles different from that of the short circuit condition in Figure 3(a). Additionally, the amplitude of the current was suppressed. The average power consumed in the load resistance is calculated from the output current and shown in Figure 4(b), indicating that the optimal load of this sample is 1 MΩ with the output power of 77 nW/cm^2^.

**Figure 5.**
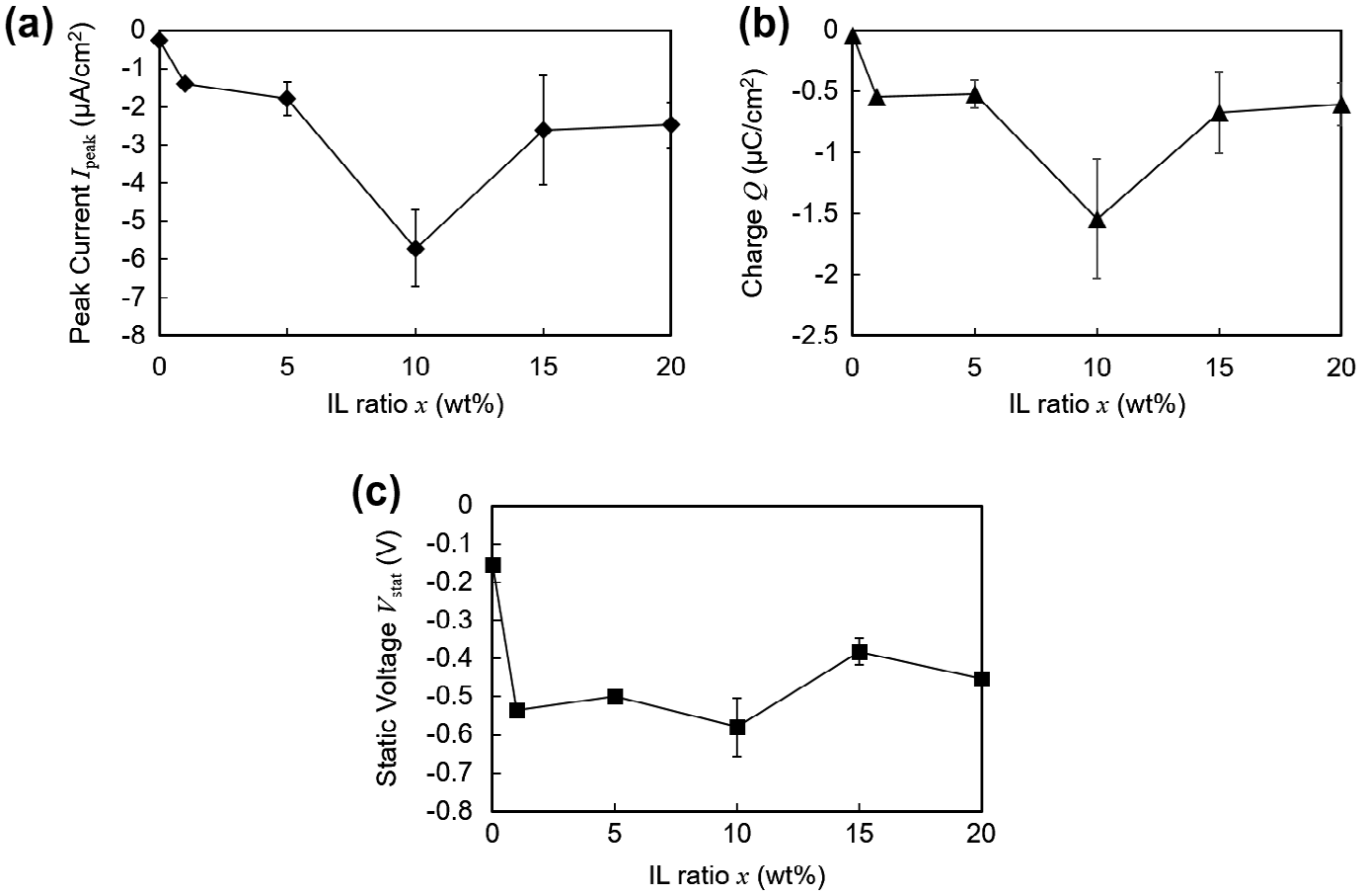
Electrical profiles plotted against varying concentrations of ionic liquid (IL) within the polymer matrix. (a) Peak current. (b) Transferred charge. (c) Static voltage.

Figure 5 shows the output dependence on the ratio of ionic liquid included in the polymer matrix. Multiple samples were prepared for each weight composition. Figure 5(a) and (b) indicates that the current output reached a maximum when the ionic liquid ratio was 10 wt%. However, it must be noted that excessive liquid was seen on the surface of the polymer when the ratio was 15 wt% or 20 wt%, indicating insufficient amount of polymer to complete solidification. On the other hand, as shown in Figure 5(c), no significant correlation was observed between the static voltage and the ionic liquid ratio. Since there is no charge transfer between the electrodes under the open-circuit voltage measurements, this suggests that the triboelectric charge at the interface is practically constant above 1 wt%. In addition, the static voltage for polymers with ionic liquid ratio above 1 wt% was more than twice that of the polymer without any ionic liquid (0 wt%). This indicates that although contact electrification occurs between the electrode and the polymer without any ions, it is enhanced when there are ions immobilized at the surface of the polymer.

**Figure 6.**
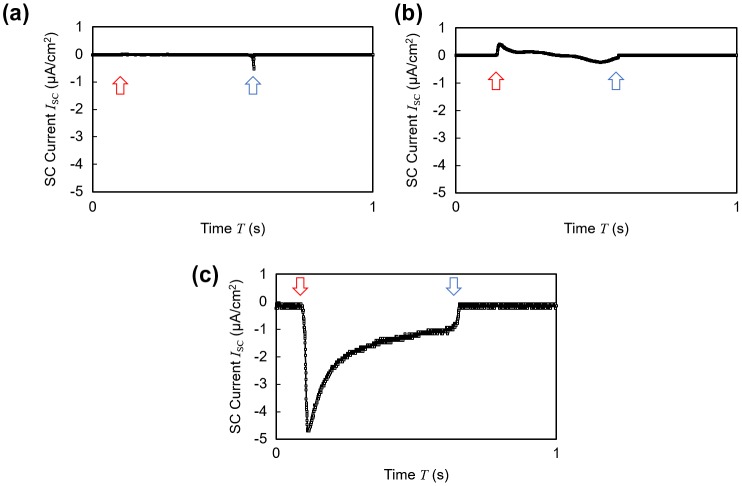
Short circuit current wave profiles of samples shown in Table 1. The red arrow indicates the contact point and the blue arrow indicates the releasing point. (a) Ionic liquid free polymer. (b) Charge-fixed polymer without polarization. (c) Surface-charge-fixed polymer with polarization.

To clarify the effect of the cations immobilized at the surface, control experiments with three different samples shown in Table 1 were conducted. When the sample did not contain ionic liquid at all as in case (a), or when the bias voltage was not applied across the electrode during polymerization although a sufficient amount of ionic liquid was mixed in the material as in case (b), no ideal EDL was formed at the interface. Thus, cases (a) and (b) yielded a smaller amount of cations immobilized at the surface as compared to case (c), which had the composition with the best performance. This difference in the surface charge amount led to the difference in both the wave profile and the amount of the generated current (Figure 6(a)–(c)).

**Table 1.** Sample conditions for control experiments.

Sample No.	Polarization (V)	IL ratio (%)
(a)	0	0
(b)	0	10
(c)	3	10

**Figure 7.**
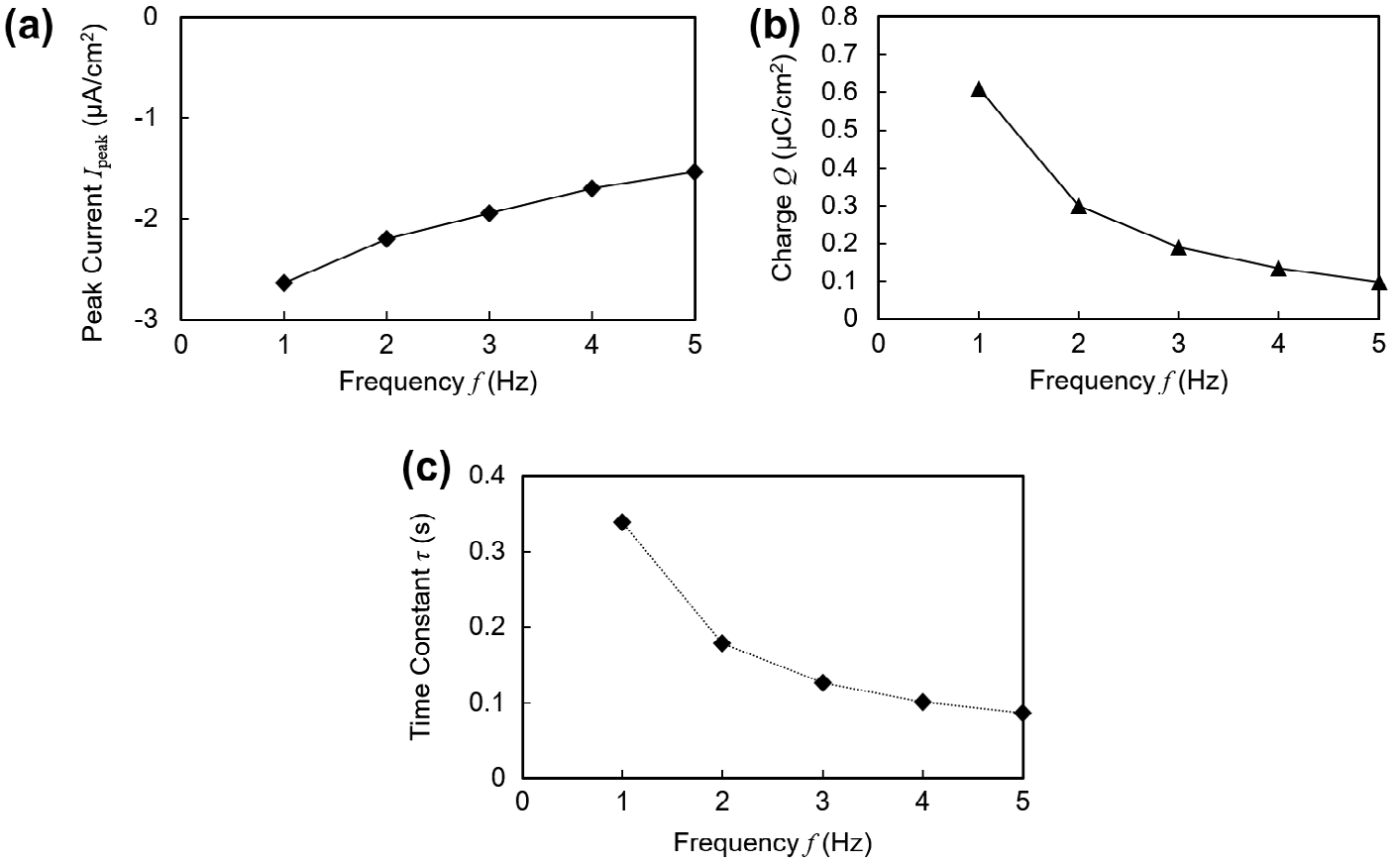
Electrical profiles plotted against varying frequencies of applied vibration. (a) Peak current. (b) Transferred charge. Charge amount is for one contact duration as shown in Figure 3(a). (c) Time constant.

In addition, the energy harvesting performance at different vibration frequencies from 1 to 5 Hz was measured. The frequency dependence of the short circuit current is plotted in Figure 7(a)–(c). Compared to the short circuit waveform under 1 Hz as shown in Figure 3(a), the peak current decreased and decayed more quickly at higher frequencies. Both the peak current in Figure 7(a) and the charge amount in Figure 7(b), as defined in Figure 3, were found to have a negative correlation with the vibration frequency. We suppose that the mechanical response of the polymer was overdamped at high vibration frequencies. In other words, at higher frequencies the next contact-separation cycle started before the mechanical structure of the polymer returned to the initial state.

Also, as shown in Figure 7(c), the time constant of the exponential decay of the short circuit current profile exhibited a decrease, leading to sharper initial current peaks at higher frequencies. This result suggests that the exponential decay of the current has a correlation with the contact and pressing speed of the electrode, since the speed is proportional to the vibration frequency.

## 6. Conclusions

We proposed a triboelectric energy harvesting system utilizing a polymer containing ionic liquid whose cations were mainly immobilized on the surface. An output power of 77 nW/cm^2^ was obtained with a load resistance of 1 MΩ at 1 Hz. The output current exceeded the μA level, which is a substantial amount when we consider target applications for IoT sensors. Given the wide range of currents achievable by varying the composition ratio, it is possible to tune the output properties of the polymer material to suit various applications that require different power. Further investigation including direct observation of the surface-charge-fixed polymer is necessary to elucidate the energy harvesting mechanism.

## Disclosure statement

No potential conflict of interest was reported by the authors.

## Funding

This work was supported by JST CREST [grant number JPMJCR15Q4].
